# Valedictory of the Year

**Published:** 1878-12

**Authors:** 


### Editorial.

### VALEDICTORY OF THE YEAR.

The third year of publication of this journal, since the organization of the Medical Press Association, is completed with the issue of this number. It has been, in every respect, the most successful and satisfactory of all the years that have passed since the issue of the first number in 1844.

This journal, when it ceased to be the organ of any medical school or set of medical men, entered upon a career of usefulness which could hardly have been anticipated. The only rivalry between physicians and between medical colleges which these pages have evinced under the present management, has been an honorable rivalry between contributors respecting the worth and value of their communications. Here have appeared, side by side, the papers of authors which, but a few years ago, would have seen the light only in medical periodicals of limited circulation and discordant interests. This happy and wholesome association has served to divorce medical politics from medical literature in Chicago, and has contributed to a cordial understanding between professional gentlemen of this city, which, by the liberal minded among them, is highly appreciated.

But the influence of this journal has been more widely felt. From the position of a provincial publication, it has advanced to become one of the national exponents of medicine in America. Its foreign exchanges, reaching hither from every quarter of the globe, reproduce, with each month, an increasingly large number of original papers which were here first published. Its editorial articles have been copied and quoted in all parts of the Union. Its foreign correspondence has been re-published, on many occa sions, with favorable comments, on this side of the Atlantic. Unsolicited endorsements of its high character and standing have appeared in the medical publications of Great Britain, France and Germany.

With this enlargement in its aim and scope, the list of its subscribers has not only increased, but extended so as to include the residents in the most distant parts of the country. An edition is now distributed from California to Maine, and from Canada to Louisiana. As might naturally be expected from such a circulation, our list of contributors is no longer limited to the body of practitioners who have settled in the Northwest. The physicians of Massachusetts, New York and Maryland, as well as those of Wisconsin, Iowa, Minnesota, Indiana and Illinois, have aided in spreading before our readers the results of scientific thought and labor. In consequence of all this, we have to announce that during the year now completed, the publishers have been enabled to expend a large sum upon the management of the journal, which represents the surplus of its receipts over and above the amount of its necessary expense.

For these results the editors are well aware that they are indebted, less to their efforts than to the excellence of the contributions of correspondents, authors and collaborateurs. To all such they extend their congratulations and thanks, in the hope that during the year to come they may look to the same and other sources for even better results. For themselves, they engage that no efforts shall be spared to still further enlarge the scope and usefulness of the CHICAGO MEDICAL JOURNAL AND EXAMINER.

No more important change has been made in the conduct of this journal during the past year, than the adoption of the metric system in its pages. In this important movement, we took a step in advance of all the other medical publications in this country. It is a step for which we have received much credit and many congratulations — a step which we have never had occasion to regret. Without urging any physician to make abrupt changes in his mode of prescribing, we thought that a wise and useful measure, which would gradually make our readers and correspondents familiar with the system, at least in its written forms. It cannot be doubted that metric measures, already extensively employed among scientists of all nations, legalized by act of congress, and urgently recommended to the profession of this country by its national association, will eventually be used, at least by physicians and druggists, to the exclusion of all other standards. In anticipation of this desirable end, the JOURNAL AND EXAMINER is content if its pages shall contribute to the result by their monthly exposition of the nomenclature, meaning and value of the terms of the metric system of weights and measures.

For the CHICAGO MEDICAL JOURNAL AND EXAMINER, the retrospect of the past year is therefore a review of its successes; its future is assuredly full of promise. In that future it shall not fail to contribute its share to the work of elevating the standards of medical education, diffusing a knowledge of the results of scientific research the world over, and supporting all measures which look to the advancement of the best interests of the medical profession.

